# Novel role of zonulin in the pathophysiology of gastro-duodenal transit: a clinical and translational study

**DOI:** 10.1038/s41598-021-01879-y

**Published:** 2021-11-17

**Authors:** Enid E. Martinez, Jinggang Lan, Takumi Konno, Alba Miranda-Ribera, Maria Fiorentino, Nilesh M. Mehta, Alessio Fasano

**Affiliations:** 1 Department of Anesthesiology, Critical Care and Pain Medicine, Boston Children’s Hospital, Boston, MA USA; 2 Department of Pediatrics, Mucosal Immunology and Biology Research Center, Massachusetts General Hospital, Boston, MA USA; 3 Department of Pediatrics, Division of Pediatric Gastroenterology and Nutrition, Massachusetts General Hospital for Children, Boston, MA USA; 4 Harvard Medical School, Boston, MA USA; 5 Massachusetts General Hospital-East, 16th Street, Building 114 (M/S 114-3503), Charlestown, MA 02114-4404 USA

**Keywords:** Gastrointestinal models, Gastrointestinal models, Paediatric research, Translational research

## Abstract

We examined the relationship between zonulin and gastric motility in critical care patients and a translational mouse model of systemic inflammation. Gastric motility and haptoglobin (HP) 2 isoform quantification, proxy for zonulin, were examined in patients. Inflammation was triggered by lipopolysaccharide (LPS) injection in *C57Bl/6* zonulin transgenic mouse (Ztm) and wildtype (WT) mice as controls, and gastro-duodenal transit was examined by fluorescein-isothiocyanate, 6 and 12 h after LPS-injection. Serum cytokines and zonulin protein levels, and zonulin gastric-duodenal mRNA expression were examined. Eight of 20 patients [14 years, IQR (12.25, 18)] developed gastric dysmotility and were HP2 isoform-producing. HP2 correlated with gastric dysmotility (r = − 0.51, CI − 0.81 to 0.003, p = 0.048). LPS injection induced a time-dependent increase in IL-6 and KC-Gro levels in all mice (p < 0.0001). Gastric dysmotility was reduced similarly in Ztm and WT mice in a time-dependent manner. Ztm had 16% faster duodenal motility than WT mice 6H post-LPS, p = 0.01. Zonulin mRNA expression by delta cycle threshold (dCT) was higher in the stomach (9.7, SD 1.4) than the duodenum (13.9, SD 1.4) 6H post-LPS, p = 0.04. Serum zonulin protein levels were higher in LPS-injected mice compared to vehicle-injected animals in a time-dependent manner. Zonulin correlated with gastric dysmotility in patients. A mouse model had time-dependent gastro-duodenal dysmotility after LPS-injection that paralleled zonulin mRNA expression and protein levels.

## Introduction

Gastric dysmotility affects 40–80% of critically ill patients^1–4^. It has been associated with difficulties in providing optimal enteral nutrition, longer length of stay, pulmonary infections and mortality^1,3,5,6^. Recently, zonulin has been reported to be associated with gastric dysmotility in critical illness^4,7^. Zonulin is a family of structurally and functionally related proteins, with pre-haptoglobin (HP) 2, the precursor protein to the mature HP2 isoform*,* being the first identified member of this family^8^. In humans, pre-HP2 is only expressed in people who carry the HP2 allele^9^. In animal and in-vitro models it has been demonstrated that zonulin plays a critical role in gastrointestinal (GI) disease by reversibly disassembling the epithelial tight junction. Human studies have demonstrated the resulting pathology from loss of epithelial barrier associated with zonulin in inflammatory conditions such as celiac disease and diabetes mellitus^10–12^. Research on zonulin and GI motility, however, is limited, with no studies reported in translational models and two human studies reporting an association between zonulin and GI motility, which had divergent results^4,7^. However, increased trans-epithelial trafficking due to loss of barrier integrity in the setting of zonulin upregulation may alter the GI milieu that regulates GI motility. Therefore, developing a mouse model to study zonulin in GI motility is needed.

We present here an integrated clinical-translational model that focuses on pre-HP2, referred to as zonulin, and provides more insight into the relationship between zonulin and GI motility in health and disease. First, we examined the relationship between the presence of the HP2 allele and the HP2 isoform, as a proxy for pre-HP2 also known as zonulin, and gastric motility in patients who underwent surgery and required critical care. Then in a zonulin transgenic mouse (Ztm) homozygous for the HP2 allele, we examined for GI transit under conditions of systemic inflammation^9,13–15^. This translational mouse model will advance our understanding of the pathways by which zonulin is associated with GI dysmotility in the context of inflammation.

## Results

### Patient protocol

**Figure 1:**
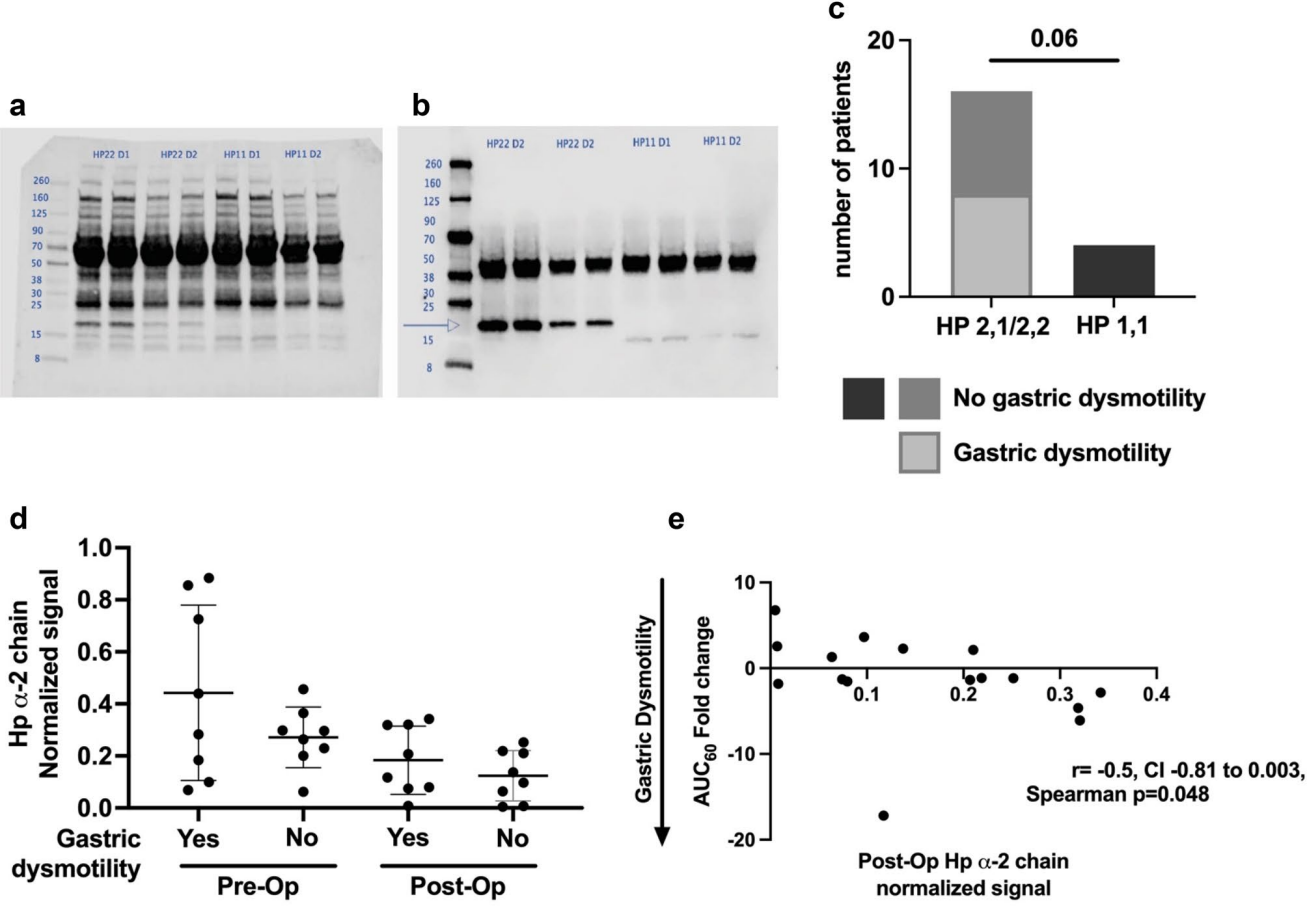
Haptoglobin (HP) α-2 chain and gastric dysmotility in patients. Panels (**a**) and (**b**) are representative Western Blots for the HP α-2 chain. Panel (**a**) is the total protein staining for quantification using LI-COR’s Revert 700 Total Protein Stain. Panel (**b**) is the corresponding membrane incubated with the primary antibody for HP. Three potential bands are expected, a ~ 45 kDa band for the β-chain that is present in all HP types and two potential α-chains, a ~ 8.9 kDa α-1 chain and a ~ 16 kDa α-2 chain. Subjects were HP typed, as designated above the lanes, based on the presence of the two potential α chains. The α-2 chain, when present, is also noted by an arrow. All subject samples were run in duplicate and LI-COR Chameleon Duo ladder was utilized for all membranes. Membranes were read in the LI-COR Odyssey. Panel (**c**) Distribution of the HP isotypes. Sixteen subjects carried at least one copy of the α-2 chain and 4 subjects carried no α-2 chain copies. Eight subjects developed post-operative gastric dysmotility, all of whom carried at least 1 copy of the HP α-2 chain. Panel (**d**) Normalized signal for the HP α-2 chain distribution. Panel (**e**) Higher normalized signal for the HP α-2 chain correlated to greater decrease in the area under the curve at 60 min (AUC60) post-operatively compared to pre-operative values in subjects undergoing surgery and requiring critical care.

Demographic and clinical characteristics of the enrolled cohort have been previously published, all other patient results have never been published^4^. In summary, 20 patients completed the study with a median age of 14 years (12.25, 18), 12 of 20 were female, and 8 of 20 developed post-operative gastric dysmotility (Table 1). Haptoglobin typing was examined by Western Blot. Figure 1a,b depict representative Western Blots showing both the total protein stain and the HP target band. The distribution of HP isotypes (HP1-1, 2-1 or 2-2) was not statistically different between our study cohort and population prevalence studies, Chi-square test p = 0.567, Table 2^16,17^. In this cohort, there was a trend towards greater gastric dysmotility in patients who were HP2-1 or HP2-2 compared to patients who were HP1-1 (Fisher’s exact test p = 0.06) (Fig. 1c). We determined the normalized signal for the HP2 isoform using quantitative Western Blot. The mean ± standard deviation of the normalized signal for the HP2 isoform was greater both pre-operatively (0.44 ± 0.34) and post-operatively (0.18 ± 0.13) in patients who developed post-operative gastric dysmotility compared to respective pre-operative (0.27 ± 0.11) and post-operative (0.12 ± 0.1) values for patients that did not develop gastric dysmotility (Fig. 1d). In zonulin-producing patients (HP2-1/2-2) there was an inverse correlation between the post-operative normalized signal for the HP2 isoform and gastric motility measured by change in the AUC_60_ of the acetaminophen absorption test, where there was a greater decrease in post-operative gastric motility as the post-operative HP2 isoform normalized signal increased, Spearman correlation rho = − 0.51, CI − 0.81 to 0.003, p = 0.048 (Fig. 1e).

**Table 1:** Demographic and Clinical Variables for the Patient Cohort. Twenty patients were enrolled in the study. This table has been Modified from reference^4^.

Demographic and clinical variables	Median (IQR^a^) or frequency (%)
Age, years	14 (12.25, 18)
Sex, female	12/20 (60%)
Anesthesia duration (min)	661 (570, 759)
Number of patients who required post-operative vasoactive agents	7/20 (35%)
Number of patients who required post-operative mechanical ventilator support	10/20 (50%)
Length of ICU stay (days)	2 (1, 3.75)

*IQR* interquartile range represented as 25th percentile, 75th percentile; EN, enteral nutrition; ICU, intensive care unit; IQR, interquartile range.

^a^

**Table 2:** Distribution of Haptoglobin (HP) isotypes in the study cohort was compared by Chi-square to the distribution of HP isotypes from population prevalence studies. ^a^The distribution of HP isotypes for the population cohort were obtained from references^16^ and ^17^.

Patients	HP 1-1	HP 2-1	HP 2-2	Chi-square test, p
Study cohort (n = 20)	4	7	9	0.57
Population studies (n = 100)^a^	16	48	36	

### Murine model

#### Inflammation

**Figure 2:**
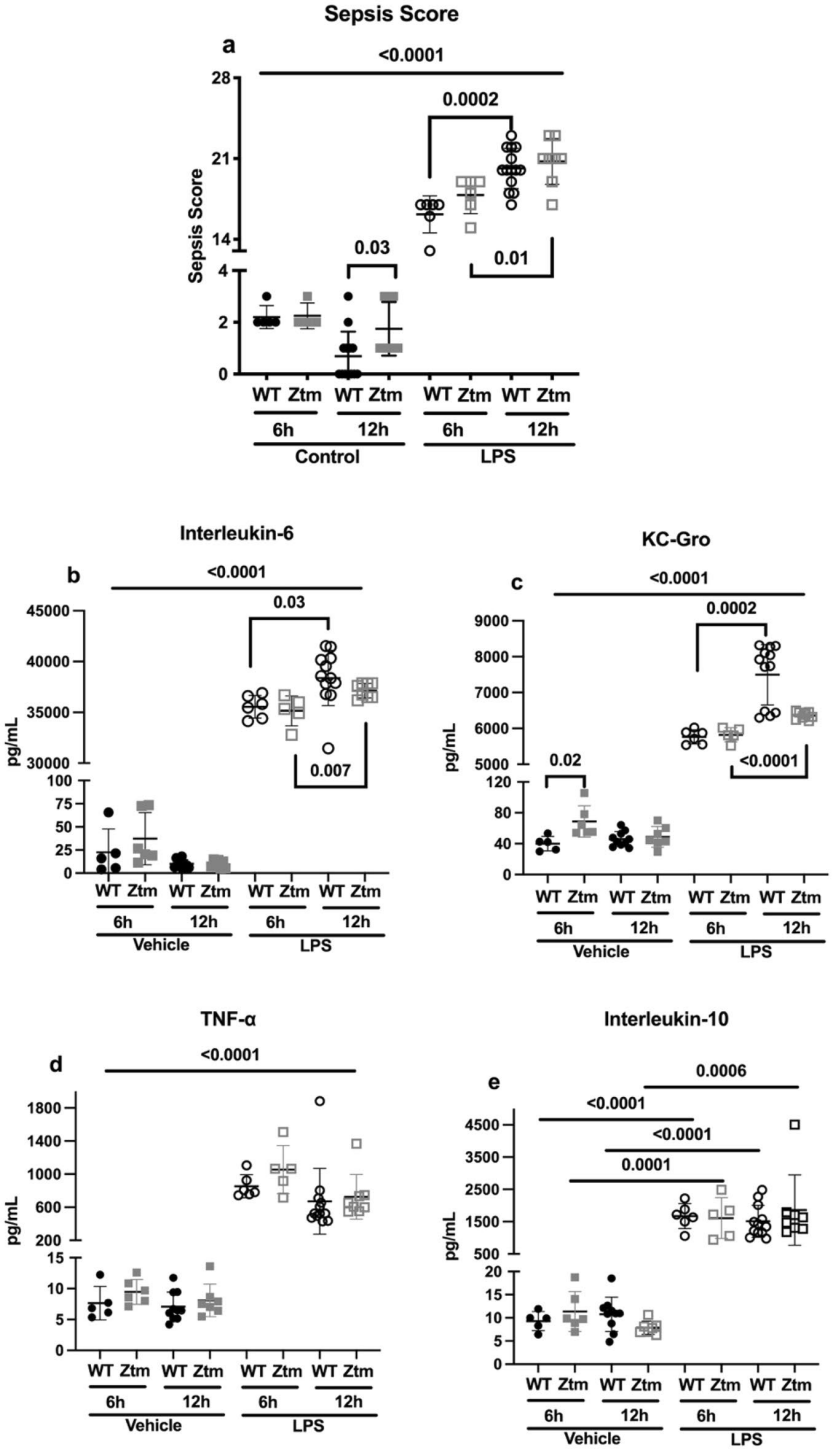
Inflammatory response to LPS 6 and 12 h after injection in wildtype, *C57Bl/6* (WT) and zonulin transgenic mice (Ztm). Panel (**a**) Sepsis scores were adjudicated based on ref. 39, 6 or 12 h after vehicle or LPS injection. Panels (**b**–**e**) Serum levels of IL-6, KC-Gro, TNF-α, and IL-10 were measured using multiplex assay. Individual values per mouse are represented for all assays. Student *t* test, p < 0.05. Long bars represent the statistical analysis between vehicle- and LPS-injected mice within each genotype. Filled circles—WT vehicle-injected; Filled squares—Ztm vehicle-injected; Open circles—WT LPS-injected; Open squares—Ztm LPS-injected. LPS-lipopolysaccharide.

Inflammation was triggered by lipopolysaccharide (LPS) injection in *C57Bl/6* Wildtype (WT) and Zonulin transgenic mice (Ztm). Sepsis scores were higher for both the WT and Ztm mice 6 and 12 h after LPS injection compared to vehicle-injected mice, p < 0.0001 (Fig. 2a). Mean sepsis scores were greater 12 h from LPS injection compared to 6 h from LPS injection for WT and Ztm mice, (WT mice 6H = 16.2 ± 1.6 and 12H = 20.1 ± 1.7, p = 0.0002; Ztm mice 6H = 17.8 ± 1.6 and 12H = 20.8 ± 2, p = 0.01) (Fig. 2a).

Serum levels of IL-6, KC-Gro, TNF-α and IL-10 were higher in LPS-injected mice compared to vehicle-injected mice, p < 0.0001 for Fig. 2b–d and p < 0.001 for Fig. 2e. Serum IL-6 and KC-Gro levels were higher 12 h post-LPS injection compared to 6 h post-LPS injection in both WT mice [IL-6 12H = 38,386 pg/mL ± 2720 and 6H = 35,552 pg/mL ± 1122, p = 0.03 (Fig. 2b); KC-Gro 12H = 7498 pg/mL ± 843 and 6H = 5767 pg/mL ± 198, p = 0.0002 (Fig. 2c)] and Ztm mice [IL-6 12H = 37,131 pg/mL ± 718 and 6H = 35,145 pg/mL ± 1475, p = 0.007 (Fig. 2b); KC-Gro 12H = 6359 pg/mL ± 107 and 6H = 5823 pg/mL ± 196, p < 0.0001 (Fig. 2c)].

#### Gastric and duodenal transit

**Figure 3:**
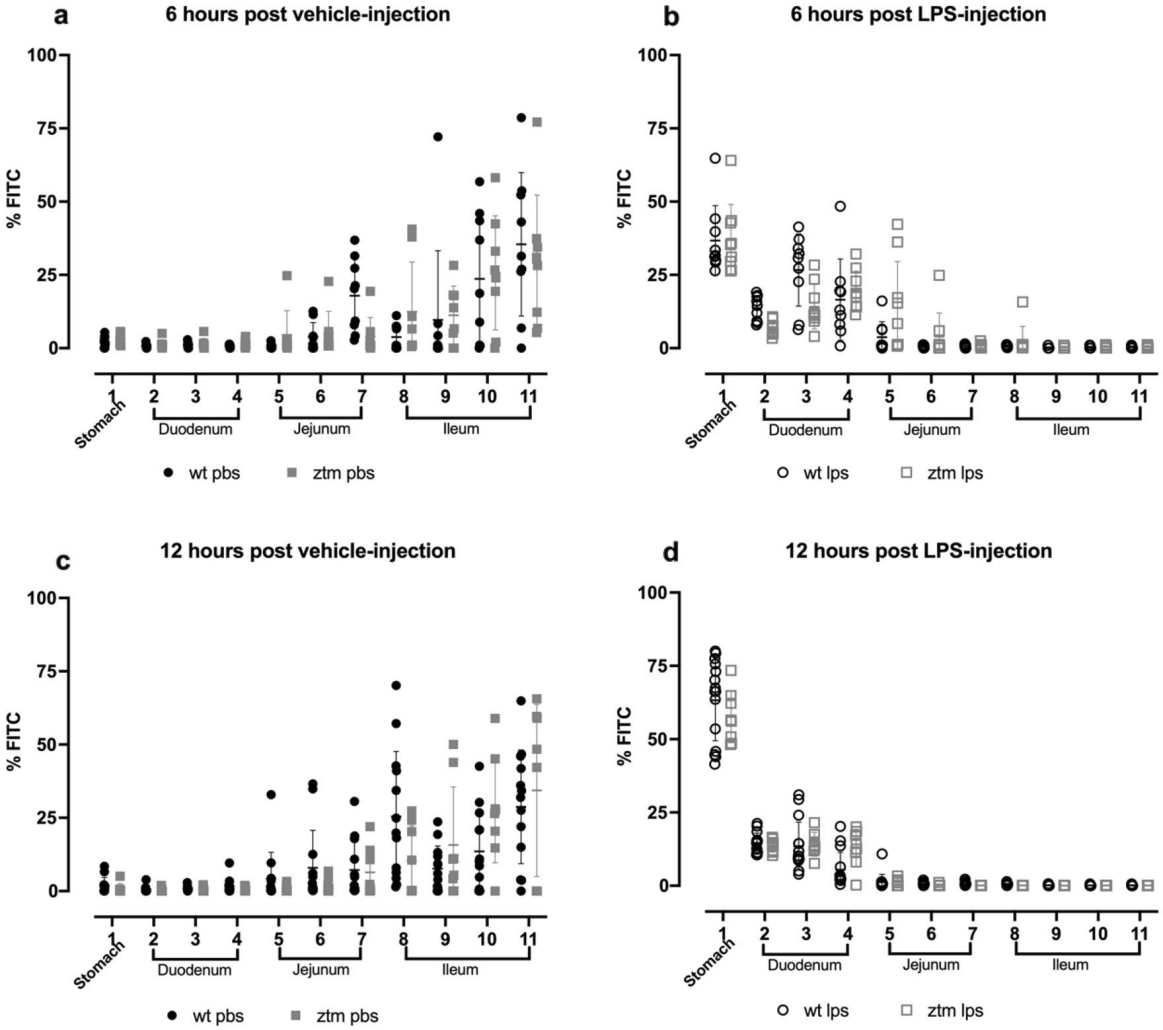
Gastric and small intestine motility measured by FITC-dextran. Mice were gavaged with FITC-dextran, and FITC-dextran signal was measured in the stomach and 10-equal segments of the small intestine at 485/535 nm wavelength 6 or 12 h after vehicle or LPS injection. Individual values per mouse are represented. Filled circles—WT vehicle-injected; Filled squares—Ztm vehicle-injected; Open circles—WT LPS-injected; Open squares—Ztm LPS-injected. LPS-lipopolysaccharide.

**Figure 4:**
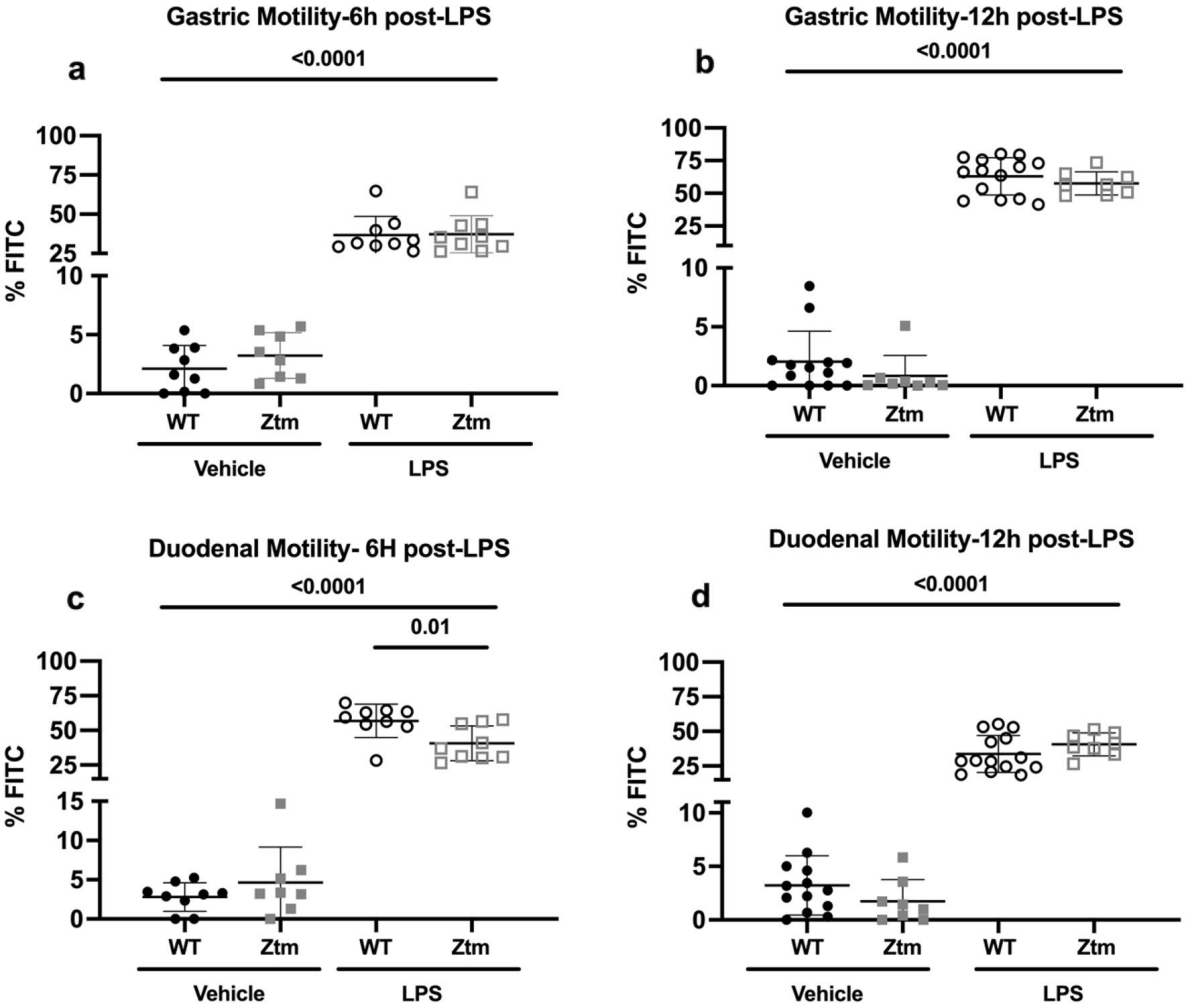
Gastric and duodenal percent of FITC-dextran. Percent FITC-dextran in the first three segments of the small intestine were summed and represent the duodenum. Individual values per mouse are represented. One-way ANOVA, p < 0.05. Long bars represent the statistical analysis between vehicle- and LPS-injected mice. Filled circles—WT vehicle-injected; Filled squares—Ztm vehicle-injected; Open circles—WT LPS-injected; Open squares—Ztm LPS-injected.

Gastrointestinal motility was measured by fluorescein-isothiocyanate-dextran (FITC-dextran) transit to examine for differences in motility between WT and Ztm mice at baseline and under conditions of inflammation. Figure 3a–d depicts the distribution of FITC-dextran percent in each segment of the stomach and small intestine in all experimental groups, whereby higher %FITC-dextran in stomach or duodenum represent slower motility. Mean percentage of FITC-dextran increased in the stomach 6 and 12 h after LPS-injection compared to vehicle-injection for all experimental groups, p < 0.0001 (Fig. 4a,b). Mean percentage of FITC-dextran in the stomach 6 h after LPS injection increased to 36.8% ± 11.9 in WT mice and 37.2% ± 11.9 in Ztm mice (Fig. 4a), and then to 63.3% ± 14.3 in WT mice and 57.6% FITC ± 8.8 in Ztm mice 12 h after LPS injection (Fig. 4b).

Mean percentage of FITC-dextran also increased in the duodenum 6 and 12 h after LPS injection compared to vehicle-injection (Fig. 4c,d). Six-hours after LPS injection Ztm mice had 16% lower percent FITC-dextran (40.6% FITC ± 12.5) in the duodenum compared to WT mice (56.9% ± 12), p = 0.003 (Fig. 4c). Twelve-hours after LPS injection, mean percentage FITC-dextran in the duodenum was 33.7% ± 13 in WT mice and 40.6% ± 8.3 in Ztm mice in the setting of greater stomach retention (Fig. 4d).

#### Zonulin mRNA expression and protein levels

**Figure 5:**
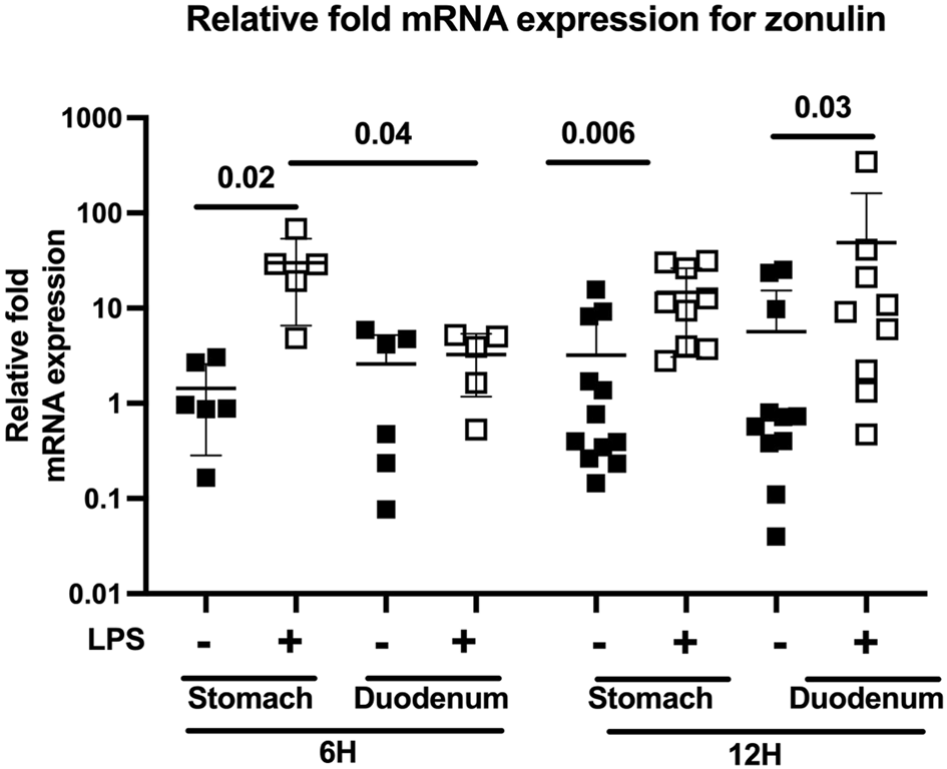
Gastric and duodenal mRNA expression of zonulin. Relative fold mRNA expression are presented for visual representation, and delta cycle threshold (dCT, target gene CT – housekeeping gene CT) values were utilized for statistical analyses using one-way ANOVA, p < 0.05. Individual values per mouse are represented. Filled squares—Ztm vehicle-injected; Open squares—Ztm LPS-injected.

Zonulin mRNA expression was measured in gastric and duodenal tissue of Ztm. mRNA expression of zonulin was greater in the stomach of Ztm mice 6 h after LPS injection and in the stomach and duodenum 12 h after LPS injection, compared to Ztm vehicle-injected mice (Stomach—6 h Ztm LPS dCT 9.7 ± 1.4 and Vehicle 14.1 ± 0.1.5, p = 0.02; Stomach—12 h Ztm LPS dCT 9.9 ± 1.4 and Vehicle 13.3 ± 2.3, p = 0.006; Duodenum—12 h Ztm LPS dCT 12.1 ± 2.8 and Vehicle 15.2 ± 3, p = 0.03) (Fig. 5). There was no difference in zonulin mRNA expression of the duodenum 6 h after LPS or vehicle injection.

Increases in zonulin mRNA expression paralleled gastroduodenal motility changes in Ztm. Specifically increases in zonulin expression (Fig. 5) and dysmotility (Fig. 4a,b) of the stomach were present 6 and 12 h after LPS injection, but increase in zonulin expression (Fig. 5) and dysmotility (Fig. 4d) of the duodenum was only present 12 h after LPS injection whereas zonulin expression (Fig. 5) was not increased 6 h after LPS injection in the setting of attenuated duodenal dysmotility (Fig. 4c).

**Figure 6:**
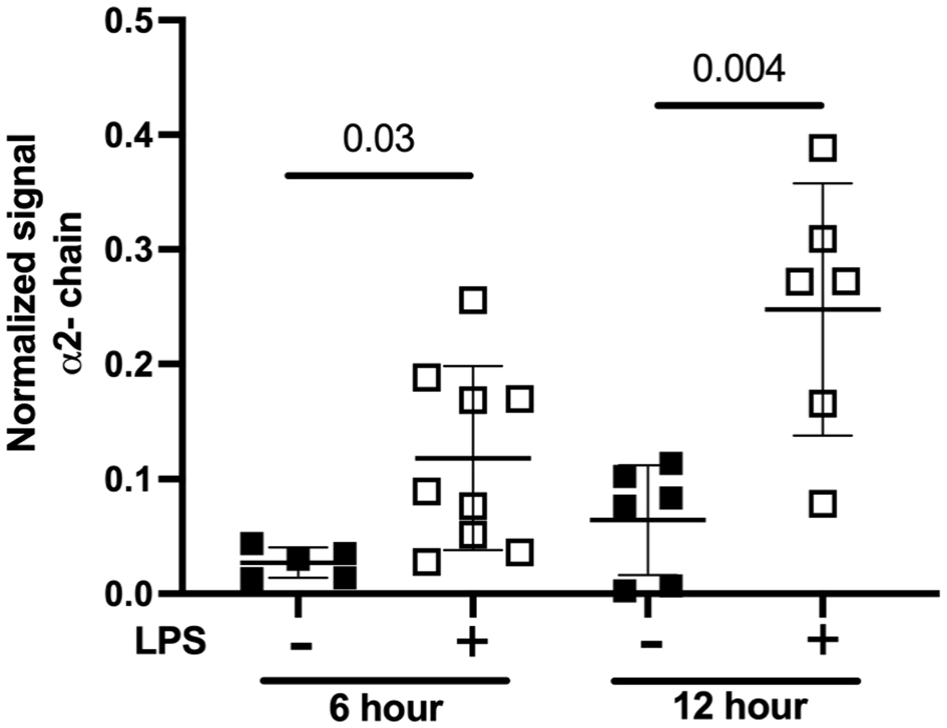
Mouse serum Haptoglobin (HP) α-2 chain, semi-quantification using Western Blot in Ztm. Filled squares—Ztm vehicle-injected; Open squares—Ztm LPS-injected. Unpaired Student *t* test, p < 0.05.

The HP2 isoform was quantified in serum from vehicle- and LPS-injected Ztm mice. The normalized signal for the HP2 isoform, proxy for zonulin, was significantly higher in LPS-injected mice compared to the vehicle-injected mice 6 and 12 h after LPS-injection (Fig. 6). The mean (± SD) normalized signal for the HP2 isoform was greater 12 h after LPS-injection (0.25 ± 0.11) compared to 6 h after LPS-injection (0.12 ± 0.08).

## Discussion

We have identified a novel relationship between zonulin and gastric motility under conditions of inflammation in a patient cohort and a transgenic mouse. Gastric dysmotility after major surgery developed in patients who carried the HP2-2 or HP2-1, zonulin-producing, isotypes, and the HP2 isoform signal correlated with decreasing gastric motility. In the translational mouse model, Ztm mice had dysmotility that was GI-segment specific and time-dependent. Stomach and duodenal changes in motility paralleled zonulin mRNA expression. Specifically, gastric dysmotility and elevated zonulin mRNA expression first developed 6 h after LPS injection. As the inflammatory cascade evolved, evident by the time-dependent increase in cytokines, both gastric and duodenal dysmotility developed with parallel increases in zonulin mRNA expression. Protein levels of the HP2 isoform also increased over time after LPS injection. This relationship between inflammation, zonulin and GI dysmotility is novel and may shed light into the contributing factors for GI motility in health and disease.

GI dysfunction, including gastric dysmotility, has been reported in 40–80% of critically ill patients^1–4^. Significant morbidities in critical care patients have been associated with GI dysfunction^1,3,5,6^. Although zonulin has been implicated in multiple conditions known for GI dysmotility, studies examining the role of zonulin on GI motility have been limited^11,12,18^. In a cohort of critically ill adults, zonulin, as a marker for disrupted epithelial barrier, was found to be higher in patients who had gastric dysmotility^7^. We previously reported in a cohort of pediatric patients who underwent major surgery and required critical care that zonulin levels decreased as post-operative gastric motility decreased^4^. These studies were completed using an ELISA for the broad family of zonulin peptides, and was therefore not specific to pre-HP2. We have now shown, by examining the HP2 isoform, that indeed only patients who carried the HP2 isoform developed gastric dysmotility and these patients had greater zonulin levels both before and after surgery compared to patients without post-operative gastric dysmotility. We also show that the HP2 isoform signal correlated with post-operative gastric dysmotility. These results reconcile the difference in previous studies, demonstrating that although zonulin levels overall decrease post-operatively, patients who develop post-operative gastric dysmotility start with and retain higher post-operative serum zonulin levels than patients who do not develop post-operative gastric dysmotility.

Examining further the relationship between zonulin and GI motility in patients is limited by lack of tissue, therefore developing a translational model is key to overcoming the limitations of clinical research in a vulnerable patient population. We have described here a translational mouse model in which we examine the relationship between inflammation, zonulin and GI motility using Ztm. In this model, LPS injection resulted in a typical inflammatory response with increases in serum IL-6, KC-Gro, TNF-α, and IL-10, that was similar between Ztm and WT mice and parallels the inflammatory response of human subjects^19,20^. KC-Gro also increased after LPS injection in all mice groups, as would be expected, though to a lesser degree 12 h after LPS injection in Ztm mice compared to WT mice^20^. KC-Gro also known as CXCL1, is a chemokine released by macrophages to promote neutrophil recruitment^20^. KC-Gro has not been described in models of zonulin expression but its expression has been reported to be suppressed in models of LPS pre-treatment^21^. Therefore, it is possible that in Ztm mice, where there is epithelial barrier leak at baseline, and likely subsequent greater LPS translocation from the microbiome, macrophages have an altered response to an LPS challenge as it relates to the inflammatory cascade.

In this mouse model we identified early gastric dysmotility with attenuated duodenal dysmotility in Ztm mice 6 h after LPS injection, which then progressed to both gastric and duodenal dysmotility 12 h after LPS injection. These changes in motility were paralleled by an early increase in zonulin mRNA expression of the stomach but not the duodenum 6 h after LPS injection, and an increase of both gastric and duodenal zonulin mRNA expression 12 h after LPS injection. This reflects a time-dependent and GI-segment specific change in zonulin mRNA expression that is associated with gastroduonenal motility changes under conditions of inflammation. Serum protein levels of the HP2 isoform also increased over time.

Zonulin has not been directly studied in GI motility, and therefore its mechanism of action is not known. Zonulin may mediate GI motility via protease activated receptors (PARs), one of the downstream receptors for zonulin activity^9^. In an in-vitro assay of mouse stomach and guinea pig taenia coli, activation of PAR1 and PAR2 by their respective synthetic agonists, resulted in relaxation of the gastric fundus^22^. Other studies have reported varied results that have been dependent on the inflammatory conditions of the model and focused on other segments of the GI tract such as the jejunum or colon, which support a GI-segment specific, differential response to inflammation in GI function^23–25^. Another possible pathway by which zonulin affects GI motility is by the modulation of the innate immune system and its downstream interaction with neuro-enteric components of the GI tract^26^. In the Ztm mouse we previously described a greater proportion of Th17 immune cells in the small intestine^15^. In vitro studies and animal models have shown intestinal macrophages express IL-17 receptors and the Th17 cell response is macrophage dependent^27,28^. In intestinal biopsies from patients with celiac disease, a condition which is partially mediated by Th17^29^, we identified an increased frequency of mucosal macrophages, and exposing monocytes to gliadin, which upregulates zonulin^30^, resulted in macrophage differentiation towards a pro-inflammatory phenotype^10^. Gastrointestinal macrophages have been associated with GI motility in animal models, particularly macrophages of the muscularis externa which interact with the enteric nervous system^26,31–34^. Therefore, it is plausible that in the Ztm mouse, zonulin enhances antigen trafficking which alters the GI immune cell population and the balance between pro-inflammatory and anti-inflammatory macrophages, resulting in a downstream effect on GI motility.

We have described results from a clinical-translational study including a patient cohort and a mouse model that support a novel role for zonulin in GI motility. Our results show a zonulin dose-dependent effect on motility which is regulated by the degree of inflammation and is specific to individual GI segments. In this study we included human HP isotype data which allowed the examination of zonulin as pre-HP2, thereby overcoming limitations from ELISA assays that examine non-specific zonulin family peptides^8^. This limitation was further mitigated by the use of the Ztm mouse model. The strength of this transgenic mouse model is that it mimics the naturally occurring HP2 variant in humans which allows for the transcription and regulation of the HP2 allele under the same environmental conditions as native HP1-carrying WT mice. This mouse model will allow for further research to examine the downstream cellular-molecular pathways by which zonulin contributes to the regulation of motility under conditions of inflammation.

## Conclusion

Zonulin expression-dependent changes in motility are present in a cohort of patients who have undergone major surgery and required critical care and in a translational model including Ztm mice. Furthermore, the Ztm model had time-dependent and GI-segment specific changes in zonulin expression and motility under conditions of inflammation. The Ztm mouse model will support future studies to further elucidate the cellular-molecular pathways underlying this novel role for zonulin in GI function.

## Methods

### Patient cohort

The details of the study design for the patient protocol have been previously described^4^. In summary, patients scheduled for elective posterior spinal fusion surgery and requiring post-operative care in a multidisciplinary pediatric intensive care unit (PICU) were enrolled^4^. This protocol was approved by the Boston Children’s Hospital (BCH) Institutional Review Board (IRB) and all study procedures were performed in accordance with BCH and the BCH IRB’s research guidelines. All enrolled patients’ parents/guardians provided informed consent, and patients provided assent when applicable.

Gastric motility using the acetaminophen absorption test, as previously published, was examined pre-operatively (study day 1) and on post-operative day 1 (study day 2)^1,4,35^. In summary, gastrically administered acetaminophen is not absorbed in the stomach but rather in the small intestine where it is subsequently metabolized in the liver and enters circulation. Therefore, the rate at which acetaminophen is emptied from the stomach is represented by the concentration of plasma acetaminophen over time using the area under the curve at 60 min (AUC_60_, µg/ml*min) determined by pharmacokinetic analysis^1,35^. Patients with 20% or greater decrease in their post-operative AUC_60_ compared to their pre-operative AUC_60_ were categorized as having post-operative gastric dysmotility. A fold change value less than 1 reflected a decrease in AUC_60_ post-operatively and was converted to negative values by the following equation: − 1/x.

In this study we newly examined the HP isotype, which predetermines a patient’s ability to produce pre-HP2, also known as zonulin^9^. Three HP isotypes, HP1-1, 2-1 and 2-2, have been described based on the presence of two alternative alleles for the α-chain subunit of HP, and zonulin, as pre-HP2, is a precursor to the HP α-2 chain subunit also known as the HP2 isoform^9,16,17^. Identification of the HP2 isoform serves as a proxy measure for zonulin, and addresses the non-specificity of ELISA assays that measure the broader zonulin family peptides^8^. Haptoglobin isotype was determined by semi-quantitative Western Blot using anti-HP antibodies (Sigma GW20080F) and the LI-COR Revert Total Protein Normalization Protocol^36^. All patient samples were run in duplicate. Protein was quantified using the Pierce BCA kit (ThermoFisher Scientific, Rockford, IL) per manufacturer’s instructions. Serum samples were then mixed with sample buffer containing beta-mercaptoethanol and sodium dodecyl sulfate (SDS). Serum sample volume was adjusted and diluted in distilled water (dH_2_O) to load equal micrograms (μg) of protein per sample, and total final volume including serum sample, sample buffer and dH_2_O was equal for all samples. Samples were then denatured at 99 °C for 5 min, loaded into 4–20% tris–glycine gels with the LI-COR Chameleon Duo ladder and after gel electrophoresis transferred onto PVDF membranes. PVDF membranes were rinsed in dH_2_O, dried for 45 min to allow for settling of all transferred proteins and the membrane was reactivated in a methanol wash for 1 min on a shaker and rinsed with dH_2_O. Membranes were incubated in LI-COR’s Revert 700 Total Protein Stain and read per manufacturer’s instructions on LI-COR Odyssey infrared scan (LI-COR Biosciences)^36^. The membranes were then blocked with 5% non-fat milk in Tris buffered saline (TBS), and incubated overnight at 4 °C with primary antibody (1:5000) prepped in TBS-Tween 0.1% (TBST). The membranes were washed three times with TBST and incubated for 1 h (h) at room temperature with secondary antibody (1:20,000; Li-COR Biosciences) prepped in TBST + 0.02% SDS. Western blot signal was visualized in the 800 channel using the LI-COR Odyssey infrared scan (LI-COR Biosciences). As previously published, HP consists of a ~ 45 kDa β-chain that is present in all HP types and two potential α-chains, a ~ 8.9 kDa α-1 chain and a ~ 16 kDa α-2 chain making up the three isotypes^17^. For relative quantification of the HP2 isoform containing the α-2 chain, the HP α-2 chain signal was controlled for the total protein signal from LI-COR’s Revert 700 Total Protein Stain (LI-COR Biosciences) using LI-COR’s Empiria Studio software and reported as normalized signal^36^. Figure 1 includes a representative Western Blot including the membrane with the LI-COR’s Revert 700 Total Protein Stain and the target band.

### Mouse model

#### Animals

Adult (8–12 week old) Ztm, generously donated by Dr. Andrew Levy, and wildtype (WT) *C57Bl/6* mice from an in-house colony maintained at the Massachusetts General Hospital (MGH) were used for these experiments^14,15^. Wildtype mice carry only the HP1 allele and therefore do not produce zonulin, natively. The Ztm mouse has duplications within the murine HP1 allele to generate the HP2 allele, which recapitulates the human HP2 allele variant and turns the Ztm mouse into a pre-HP2, i.e. zonulin, producer^14^. All mice were housed in the same facility and maintained per standard conditions as previously described^15^. This animal protocol was approved by the MGH Institutional Animal Care and Use Committee (IACUC) and all procedures were performed in accordance with the MGH IACUC. ARRIVE guidelines were followed. All mice were 8–12 weeks at the time of the experiments. Male and female mice were included. The mean ± SD weight of the mice groups were 24.1 g ± 3.3 for WT vehicle-injected, 23.0 g ± 3.9 for WT LPS-injected, 22.6 g ± 3.8 for Ztm PBS-injected, and 23.1 g ± 3 for Ztm LPS-injected, one-way ANOVA p = 0.6. Weights were obtained 24–48 h prior to the experiment. All mouse experiments were run at a minimum in triplicate.

#### Experimental conditions

Mice were intraperitoneally (IP) injected with lipopolysaccharide (LPS) or 1% Phosphate Buffered Saline (PBS) as vehicle. *Escherichia* *coli* LPS O111:B4 (Sigma-Aldrich L3012) was reconstituted in sterile 1% PBS. Mice were weighed and 10 mg/kg of LPS or equivalent volume of vehicle were administered via intraperitoneal (IP) injection 6 or 12-h prior to GI motility testing, based on previously published studies^37^. Inflammation was confirmed by a sepsis score and serum cytokine levels^15,38^. Mice were scored either 6 h or 12 h after LPS injection per protocol. Serum cytokine levels were measured using MSD V-Plex Proinflammatory Panel kit (Rockville, Maryland) per manufacturer’s instructions. Mice were fasted from solids from time of injection (6 or 12 h) and from water for 1 h prior to euthanasia. Mice were euthanized per IACUC guidelines with isoflurane.

#### Transit testing

Gastro-duodenal transit was tested by intraoral gavage of non-digestible fluorescein isothiocyanate-dextran (FITC-dextran) as adapted from previous publications^39,40^. Mice were gavaged intra-orally with 6 mg/kg of FITC-dextran using a standard oral gavage needle 1 h prior to euthanasia. WT mice were gavaged with FITC-dextran 4000 molecular weight (MW) for the 12-h protocol and FITC-dextran 70,000 MW for the 6-h protocol. Total amount of FITC-dextran, ng FITC/g of mouse, recovered for the whole GI tract was the same between 4000 MW or 70,000 MW FITC-dextran gavaged WT mice (one-way Anova p = 0.14). Ztm mice were gavaged with 70,000 MW FITC-dextran for all experiments, given their baseline increased epithelial barrier leak^13^. Gastrointestinal tissue was isolated including the stomach and the small intestine, which was measured and divided into 10 equal segments. The stomach and each small intestine segment were flushed with 2 mL of PBS. Flush was diluted 1:100 with PBS, and FITC signal in the flush of each segment was loaded in duplicates and measured in a fluorescence reader at 485/535 nm wavelengths. FITC signal was controlled for mouse weight and the percent of FITC-dextran per segment was calculated.

#### Quantitative-polymerase chain reaction (q-PCR)

Stomach and duodenal tissue mRNA expression of zonulin were examined by q-PCR as previously published^15^. A segment of stomach and duodenum from all mouse groups was collected and immediately placed in 1 mL of TRIzol Reagent (Thermo Fisher) and homogenized. Total mRNA extraction was achieved using the Direct-zol RNA mini prep Kit (Zymo Research) following the manufacturers’ instructions. RNA concentrations and A260/A280 and A260/A230 ratios were measured with the NanoDrop spectrophotometer (Thermo Scientific). Genomic DNA was removed using the DNA free Kit (Thermo Fisher) and mRNA was reverse transcribed using random hexamer primers and Maxima universal first strand cDNA synthesis kit #1661 (Thermo Fisher Scientific, Waltham, MA) according to manufacturers’ instructions. Quantitative real-time PCR was performed using PerfeCTa SYBR Green SuperMix (Quanta) using in-house designed primers based on NIH’s Primer Blast and obtained from Integrated DNA Technologies (18S-Primers 5′ Oligo AGAAACGGCTACCACATCCA 3′ Oligo CCCTCCAATGGATCCTCGTT; Zonulin-Primers 5′ Oligo GAATGTGAGGCAGATGACAG, 3′ Oligo GTGTTCACCCATTGCTTCTC)^15^. All samples within the PCR were run in duplicates including blanks. mRNA expression of the 18S housekeeping gene was not statistically different among experimental groups. The delta cycle threshold (dCT), target gene cycle threshold − housekeeping gene cycle threshold, was calculated for statistical analysis, and the relative fold gene expression is represented in Fig. 5.

#### Haptoglobin α-2 chain quantification

The HP α-2 chain, also referred to as the HP2 isoform, was quantified using the same semi-quantitative Western Blot protocol described for the human protocol except for the following steps. Mouse serum samples were protein concentrated using 50 kDa Millipore Sigma’s Amicon Ultra-0.5 mL Centrifugal Filters (Darmstadt, Germany) per manufacturer’s instructions. Protein concentrated samples were then albumin/IgG depleted using the Pierce Albumin/IgG Removal Kit and GenDEPOT PureSelect Protein G-Agarose Fast-Flow (Barker, Texas), and protein was quantified using the Pierce BCA kit (ThermoFisher Scientific, Rockford, IL) per manufacturer’s instructions. Anti-HP antibody from Sigma GW20080F was used at a 1:250 concentration. All other steps were performed identical to the human protocol.

### Statistical analysis

Statistical analyses were completed using GraphPad Prism Version 8, GraphPad Software La Jolla, California. Data are presented as mean ± standard deviation.

Baseline demographic and clinical characteristic have been previously reported, Table 1^4^. The distribution of HP isotypes between our study cohort and population prevalence studies was examined by Chi-square test^16,17^. The proportion of patients within each HP isotype with post-operative gastric dysmotility was determined. Spearman correlation analysis between the post-operative normalized signal for the HP2 isoform and gastric motility by the AUC_60_ was examined. Normalized signal for the HP2 isoform, both pre- and post-operatively, between patients who developed post-operative gastric dysmotility and those that did not, was examined.

No differences were identified between male and female mice for all experiments and therefore data are presented as aggregate. The Bartlett test was performed to examine if there was equal variance among experimental groups. If the standard deviation between experimental groups was not statistically significant a one-way ANOVA was performed. If a statistically significant difference between standard deviations was determined then independent, unpaired Student *t* tests were performed. We examined for differences in sepsis score (Fig. 2), serum cytokines (Fig. 2), gastric and duodenal transit by the percent of retained FITC-dextran (Fig. 4), stomach and duodenal zonulin mRNA (Fig. 5) and serum HP2 isoform (Fig. 6) among mouse groups. Zonulin mRNA expression statistical analyses were performed using the dCT values and Fig. 5 depicts the relative fold gene expression.

## Data Availability

Data, analytic methods and study materials will be made available to other researchers by request.

## Abbreviations

AUC: Area under the curve
FITC: Fluorescein-isothiocyanate
GI: Gastrointestinal
HP: Haptoglobin
ICU: Intensive care unit
IL: Interleukin
LPS: Lipopolysaccharide
qPCR: Quantitative polymerase chain reaction
Ztm: Zonulin transgenic mouse
WT: Wildtype
WB: Western blot

## References

[1] Martinez EE (2017). Gastric emptying in critically ill children. JPEN J. Parenter. Enter. Nutr..

[2] Mayer AP (2002). Amylin is associated with delayed gastric emptying in critically ill children. Intensive Care Med..

[3] Eveleens RD, Joosten KFM, de Koning BAE, Hulst JM, Verbruggen S (2020). Definitions, predictors and outcomes of feeding intolerance in critically ill children: A systematic review. Clin. Nutr..

[4] Martinez EE (2020). Interleukin-10 and zonulin are associated with post-operative delayed gastric emptying in critically ill surgical pediatric patients—A prospective pilot study. JPEN J. Parenter. Enter. Nutr..

[5] Mehta NM (2010). Challenges to optimal enteral nutrition in a multidisciplinary pediatric intensive care unit. JPEN J. Parenter. Enter. Nutr..

[6] Reintam Blaser A (2013). Gastrointestinal symptoms during the first week of intensive care are associated with poor outcome: A prospective multicentre study. Intensive Care Med..

[7] Greis C (2017). Intestinal T lymphocyte homing is associated with gastric emptying and epithelial barrier function in critically ill: A prospective observational study. Crit. Care.

[8] Scheffler L (2018). Widely used commercial ELISA does not detect precursor of haptoglobin2, but recognizes properdin as a potential second member of the Zonulin family. Front. Endocrinol..

[9] Tripathi A (2009). Identification of human zonulin, a physiological modulator of tight junctions, as prehaptoglobin-2. Proc. Natl. Acad. Sci. U.S.A..

[10] Serena G (2019). Intestinal epithelium modulates macrophage response to gliadin in celiac disease. Front. Nutr..

[11] Sapone A (2006). Zonulin upregulation is associated with increased gut permeability in subjects with type 1 diabetes and their relatives. Diabetes.

[12] Ciccia F, Guggino G, Rizzo A, Alessandro R, Luchetti MM, Milling S, Saieva L, Cypers H, Stampone T, Di Bendetto P, Gabrielli A, Fasano A, Elewaut D, Triolo G (2017). Dysbiosis and zonulin upregulation alter gut epithelial and vascular barriers in patients with ankylosing spondylitis. Ann. Rheum. Dis..

[13] Sturgeon C, Lan J, Fasano A (2017). Zonulin transgenic mice show altered gut permeability and increased morbidity/mortality in the DSS colitis model. Ann. N. Y. Acad. Sci..

[14] Levy AP (2007). Haptoglobin genotype is a determinant of iron, lipid peroxidation, and macrophage accumulation in the atherosclerotic plaque. Arterioscler. Thromb. Vasc. Biol..

[15] Miranda-Ribera A (2019). Exploiting the Zonulin mouse model to establish the role of primary impaired gut barrier function on microbiota composition and immune profiles. Front. Immunol..

[16] Langlois MR, Delanghe JR (1996). Biological and clinical significance of haptoglobin polymorphism in humans. Clin. Chem..

[17] Levy AP (2010). Haptoglobin: Basic and clinical aspects. Antioxid. Redox Signal..

[18] Usai-Satta P, Oppia F, Scarpa M, Giannetti C, Cabras F (2016). Delayed gastric emptying does not normalize after gluten withdrawal in adult celiac disease. Scand. J. Gastroenterol..

[19] Kopydlowski KM (1999). Regulation of macrophage chemokine expression by lipopolysaccharide in vitro and in vivo. J. Immunol..

[20] Copeland S (2005). Acute inflammatory response to endotoxin in mice and humans. Clin. Diagn. Lab. Immunol..

[21] Medvedev AE, Kopydlowski KM, Vogel SN (2000). Inhibition of lipopolysaccharide-induced signal transduction in endotoxin-tolerized mouse macrophages: Dysregulation of cytokine, chemokine, and toll-like receptor 2 and 4 gene expression. J. Immunol..

[22] Cocks TM, Sozzi V, Moffatt JD, Selemidis S (1999). Protease-activated receptors mediate apamin-sensitive relaxation of mouse and guinea pig gastrointestinal smooth muscle. Gastroenterology.

[23] Horie A (2009). Proinflammatory cytokines suppress the expression level of protease-activated receptor-2 through the induction of iNOS in rat colon. J. Vet. Med. Sci..

[24] Fernandez-Blanco JA, Hollenberg MD, Martinez V, Vergara P (2013). PAR-2-mediated control of barrier function and motility differs between early and late phases of postinfectious gut dysfunction in the rat. Am. J. Physiol. Gastrointest. Liver Physiol..

[25] Kawabata A (2001). In vivo evidence that protease-activated receptors 1 and 2 modulate gastrointestinal transit in the mouse. Br. J. Pharmacol..

[26] Muller PA (2014). Crosstalk between muscularis macrophages and enteric neurons regulates gastrointestinal motility. Cell.

[27] Barin JG (2012). Macrophages participate in IL-17-mediated inflammation. Eur. J. Immunol..

[28] Panea C (2015). Intestinal monocyte-derived macrophages control commensal-specific Th17 responses. Cell Rep..

[29] Sapone A (2010). Differential mucosal IL-17 expression in two gliadin-induced disorders: Gluten sensitivity and the autoimmune enteropathy celiac disease. Int. Arch. Allergy Immunol..

[30] Lammers KM (2008). Gliadin induces an increase in intestinal permeability and zonulin release by binding to the chemokine receptor CXCR3. Gastroenterology.

[31] Cipriani G, Gibbons SJ, Kashyap PC, Farrugia G (2016). Intrinsic gastrointestinal macrophages: Their phenotype and role in gastrointestinal motility. Cell. Mol. Gastroenterol. Hepatol..

[32] Wehner S (2007). Inhibition of macrophage function prevents intestinal inflammation and postoperative ileus in rodents. Gut.

[33] Stein K (2018). Leukocyte-derived interleukin-10 aggravates postoperative ileus. Front. Immunol..

[34] Mikkelsen HB, Larsen JO, Hadberg H (2008). The macrophage system in the intestinal muscularis externa during inflammation: An immunohistochemical and quantitative study of osteopetrotic mice. Histochem. Cell Biol..

[35] Nimmo WS, Heading RC, Wilson J, Tothill P, Prescott LF (1975). Inhibition of gastric emptying and drug absorption by narcotic analgesics. Br. J. Clin. Pharmacol..

[36] Taylor SC, Berkelman T, Yadav G, Hammond M (2013). A defined methodology for reliable quantification of Western blot data. Mol. Biotechnol..

[37] Pawlinski R (2004). Role of tissue factor and protease-activated receptors in a mouse model of endotoxemia. Blood.

[38] Shrum B (2014). A robust scoring system to evaluate sepsis severity in an animal model. BMC. Res. Notes.

[39] Suliburk JW (2005). Ketamine inhibits lipopolysacharide (LPS) induced gastric luminal fluid accumulation. J. Surg. Res..

[40] Tsukamoto T (2011). Novel model of peripheral tissue trauma-induced inflammation and gastrointestinal dysmotility. Neurogastroenterol. Motil..

